# Editorial for “Properties and Applications of Graphene and Its Derivatives”

**DOI:** 10.3390/nano12040602

**Published:** 2022-02-11

**Authors:** Jose M. González-Domínguez

**Affiliations:** Group of Carbon Nanostructures and Nanotechnology (G-CNN), Instituto de Carboquímica, ICB-CSIC, C/Miguel Luesma Castán 4, 50018 Zaragoza, Spain; jmgonzalez@icb.csic.es

Since the very first landmark report by Geim and Novoselov in 2004 on graphene [1], the interest of the scientific and technological communities for every form of this carbon-based nanomaterial has only grown and grown. In 2019, the hype for graphene turned fifteen years old and, after having experienced an intense infancy (with impressive results at laboratory scales in a myriad of different application fields), experts say that the following 15 years should be oriented towards its commercialization and day-to-day uses [2]. For this, some aspects are crucial and need to be understood, such as standardization or safety issues [2,3]. The broad family of graphene nanomaterials (including graphene nanoplatelets, graphene oxide, graphene quantum dots, and many more), go beyond and aim higher than mere single-layer (‘pristine’) graphene; thus, their potential has sparked the current Special Issue. In it, 18 contributions (distributed in 14 research articles and 4 reviews) have probably portrayed the most interesting lines as regards future and tangible uses of graphene derivatives.

Works on the fabrication of graphene, nanomaterials have appeared; for example, the electrochemical synthesis of highly oxidized graphene oxide (GO), by Díez-Pascual and co-workers [4], who aimed at maximizing its oxidation degree by tuning the electrochemical synthesis parameters. In another reference work, Lee et al. explored a somehow opposed concept: obtaining low-defect graphene, in this case by a tailored liquid-phase shear exfoliation of graphite [5]. Both papers demonstrate the potential up-scalability of their respective fabrication processes, and the utility of the as-made graphene-based nanomaterials, such as for instance, electrode modification for enhanced sensing purposes [5].

Some published papers in this Special Issue focus on the performance peculiarities of graphene-based nanomaterials in specific contexts. For example, Torres and co-workers have studied the effect of the reduction temperature in the electrochemical performance of reduced-GO-based nanofibers obtained by a hydrothermal method [6]. The authors found a promising 16-fold increase in capacitance as compared to the preceding GO nanofibers. Another entry on reduced GO has been reported by Toulbe et al. [7], who have found that this nanomaterial is capable of inhibiting the photodegradation of α-lipoic acid (an antioxidant) in the presence of Au and Ag nanoparticles, with important implications in pharmaceutical compounds. As a complementary insight into both of the aforementioned studies, Tene and co-workers present an elegant study on how the drying conditions of GO critically affect its structure and nature of defects [8]. The same authors report also on the environmental advantages of reduced GO by efficiently removing water pollutants [9].

In this Special Issue, there is also room for chemically modified or assembled graphene nanomaterials, such as those presented by La et al. [10], who have obtained oleic acid-modified graphene nanoplatelets showing excellent dispersion stability and tribological performance in lubricant oil. On the other hand, Pruna and co-workers present GO-based cryogels modified with amine moieties [11]. In particular, for ethylenediamine-functionalized cryogels, the authors found a promisingly high CO_2_ uptake, with a view to future CO_2_ capture technologies. If cryogels are a good example of an assembled graphene-based scaffold, and given the high interest for such 3D ensembles, the readers cannot miss the review paper presented by Bellet et al. [12]. It deals with this kind of structure; in this case, oriented towards biomedical applications (regenerative medicine), covering many aspects, from toxicity to applications, and structure–properties relationships.

However, the most reported field of application in graphene-based materials within this Special Issue, nearly half of the total contributions, has definitely been the nanocomposites one. Different nanocomposite studies for structural applications are reported, such as that authored by Sánchez-Romate et al. [13], dealing with graphene nanoplatelet-based epoxy composites with exhaustive electromechanical and electrothermal characterizations; or also two review papers with particular relevance on construction and building materials: one by Ikram et al., which covers the state-of-the art of graphene nanomaterials for water-based drilling fluids [14], and the other review by Rehman et al., gathering significant advances in graphene–cement composites [15]. Regarding electric or electronic applications of graphene nanocomposites, there are also excellent pieces of work in this Special Issue, such as that from Rendón-Patiño and co-workers (dealing with graphene–MoS_2_ heterostructures with excellent catalytic activity towards H_2_ and O_2_ evolution reactions) [16], or the work by Rodríguez-Mas et al., who have successfully inserted a conductive polymer-coated reduced GO layer in an organic-based LED, increasing its current density [17]. The great potential of GO to stand as a versatile adjuvant in advanced applications is herein embodied by the works of Petris et al. (GO–silicophosphate compounds for the optical limiting of femtosecond lasers) [18], and that of González-Domínguez and co-workers (dealing with aqueous inks based on GO–nanocellulose hybrids with potential to be applied in electrode manufacturing) [19].

Last, but not least, this Special Issue contains a unique review paper on the properties and applications of Graphyne derivatives [20], emerging as the next stage of the current graphene state-of-the-art.
